# Differential Growth of *Francisella tularensis*, Which Alters Expression of Virulence Factors, Dominant Antigens, and Surface-Carbohydrate Synthases, Governs the Apparent Virulence of *Ft* SchuS4 to Immunized Animals

**DOI:** 10.3389/fmicb.2017.01158

**Published:** 2017-06-22

**Authors:** Kristen M. Holland, Sarah J. Rosa, Kolbrun Kristjansdottir, Donald Wolfgeher, Brian J. Franz, Tiffany M. Zarrella, Sudeep Kumar, Raju Sunagar, Anju Singh, Chandra S. Bakshi, Prachi Namjoshi, Eileen M. Barry, Timothy J. Sellati, Stephen J. Kron, Edmund J. Gosselin, Douglas S. Reed, Karsten R. O. Hazlett

**Affiliations:** ^1^Department of Immunology and Microbial Disease, Albany Medical CollegeAlbany, NY, United States; ^2^Department of Biomedical Sciences, Midwestern UniversityDowners Grove, IL, United States; ^3^Department of Molecular Genetics and Cell Biology, University of ChicagoChicago, IL, United States; ^4^Trudeau InstituteSaranac Lake, NY, United States; ^5^Department of Microbiology and Immunology, New York Medical CollegeValhalla, NY, United States; ^6^School of Medicine, University of MarylandBaltimore, MD, United States; ^7^Center for Vaccine Research, University of PittsburghPittsburgh, PA, United States

**Keywords:** tularemia immunization, bacterial adaptation, virulence factor, bacterial capsules, bacterial media

## Abstract

The gram-negative bacterium *Francisella tularensis* (*Ft*) is both a potential biological weapon and a naturally occurring microbe that survives in arthropods, fresh water amoeba, and mammals with distinct phenotypes in various environments. Previously, we used a number of measurements to characterize *Ft* grown in Brain-Heart Infusion (BHI) broth as (1) more similar to infection-derived bacteria, and (2) slightly more virulent in naïve animals, compared to *Ft* grown in Mueller Hinton Broth (MHB). In these studies we observed that the free amino acids in MHB repress expression of select *Ft* virulence factors by an unknown mechanism. Here, we tested the hypotheses that *Ft* grown in BHI (BHI-*Ft*) accurately displays a full protein composition more similar to that reported for infection-derived *Ft* and that this similarity would make BHI-*Ft* more susceptible to pre-existing, vaccine-induced immunity than MHB-*Ft*. We performed comprehensive proteomic analysis of *Ft* grown in MHB, BHI, and BHI supplemented with casamino acids (BCA) and compared our findings to published “omics” data derived from *Ft* grown *in vivo*. Based on the abundance of ~1,000 proteins, the fingerprint of BHI-*Ft* is one of nutrient-deprived bacteria that—through induction of a stringent-starvation-like response—have induced the FevR regulon for expression of the bacterium's virulence factors, immuno-dominant antigens, and surface-carbohydrate synthases. To test the notion that increased abundance of dominant antigens expressed by BHI-*Ft* would render these bacteria more susceptible to pre-existing, vaccine-induced immunity, we employed a battery of LVS-vaccination and S4-challenge protocols using MHB- and BHI-grown *Ft* S4. Contrary to our hypothesis, these experiments reveal that LVS-immunization provides a barrier to infection that is significantly more effective against an MHB-S4 challenge than a BHI-S4 challenge. The differences in apparent virulence to immunized mice are profoundly greater than those observed with primary infection of naïve mice. Our findings suggest that tularemia vaccination studies should be critically evaluated in regard to the growth conditions of the challenge agent.

## Introduction

*Francisella tularensis* (*Ft*) is an extremely infectious gram-negative bacterium that is readily aerosolized. Inhalation of this select agent can lead to pulmonary tularemia which has a mortality rate of ~35% in the absence of treatment. Reportedly, antibiotic-resistant strains of this bacterium were developed by at least one nation's biological weapons program (Oyston et al., 2004). The risk of such an agent being maliciously employed, in concert with the current lack of a licensed tularemia vaccine, has prompted intense interest in *Ft*. This pathogen is naturally-occurring in diverse environments including warm- and cold-blooded hosts (Oyston et al., 2004) and has both intracellular (replicative) and extracellular (transmissive) phases (Abd et al., 2003; Forestal et al., 2007; Yu et al., 2007; Bar-Haim et al., 2008; Thelaus et al., 2009). Mounting evidence indicates that as *Ft* progresses through the intracellular cycle (phagocytosis, phagosomal escape, logarithmic cytoplasmic replication, induction of autophagic vacuoles, and cellular egress) the bacterium adapts by modulating expression of many *Ft* genes (de Bruin et al., 2007; Chong et al., 2008; Wehrly et al., 2009).

A number of studies have examined the role of relevant environmental cues such as temperature (Horzempa et al., 2008), iron (Deng et al., 2006; Lenco et al., 2007), and amino acid concentration (Hazlett et al., 2008; Zarrella et al., 2011) on bacterial gene expression. Horzempa et al. found that ~11% of the *Ft* transcriptome was differentially-expressed in *Ft* LVS grown at 26 and 37°C in Chamberlain's defined medium (CDM; Chamberlain, 1965; Horzempa et al., 2008). These genes included heat-shock proteins and those involved in amino-acid metabolism but, with one exception, did not include members of the Francisella Pathogenicity Island (FPI) which are required for infection of both mammalian and non-mammalian hosts. Deng et al. compared *Ft* LVS grown in Mueller-Hinton Broth (MHB) that was iron-replete or -deficient and identified 80 Fe-responsive genes (Deng et al., 2006). The most strongly induced (10–20 fold change) genes were those involved with synthesis and utilization of Fe-chelating siderophores (Deng et al., 2006; Sullivan et al., 2006; Ramakrishnan et al., 2008). In this study, more modest impacts (~2- to 3-fold) were noted for the remaining 75 genes including chaperones, ribosomal proteins, and select FPI proteins such as members of the Intracellular growth locus (Igl). Within the mammalian environment, the bacterium is thought to be exposed to fluctuations in growth phase and levels of nutrients such as amino acids which are ~20 times higher in the host cell cytoplasm than in the extracellular milieu (Bergstrom et al., 1974). Guina et al. observed that *F. novicida* (*Fn*) mutants lacking a key transcriptional regulator (MglA) have a marked survival defect upon entering stationary phase in a restrictive environment (MHB). In this study, the defects were linked to increased susceptibility to nutrient starvation and oxidative stress (Guina et al., 2007). The impact of stationary phase on the proteome of *Ft* has been confirmed with both the live vaccine strain (LVS) and the human-virulent strain SchuS4 (S4) (Lenco et al., 2009). Interestingly, FPI-induction in CDM-grown, stationary phase LVS and S4 is lower (~2-fold) than that reported for *Ft* LVS and S4 grown *in vivo* which show an FPI-induction of ~4- to 5-fold (Twine et al., 2006a; Wehrly et al., 2009; Bent et al., 2013; Thomas-Charles et al., 2013). We have used growth of *Ft* LVS and S4 in MHB and BHI broth as FPI-restrictive and FPI-permissive models respectively. By using BHI supplemented with casamino acids (BCA) we found that Igl induction in BHI-*Ft* is sensitive to the concentration of free amino acids (Hazlett et al., 2008).

The mutability of *Ft* presents a challenge to vaccine development as the nature of the challenge inoculum (with potentially distinct antigenic compositions) can vary widely. Upon entering the mammalian environment, the surviving inoculated bacteria will presumably adapt regardless of its prior history (i.e., phenotype). However, the window of time required for complete bacterial adaptation and the impact of this window on infection and immunization outcomes is still a developing field of inquiry. Cherwonogrodzky et al. first reported that the virulence of *Ft* LVS for naïve mice could be increased by repeated passage of the bacterium in CDM to promote accumulation of capsular material (Cherwonogrodzky et al., 1994). Loegering et al. demonstrated that *Ft* LVS passed through host cells were less pro-inflammatory to uninfected macrophages (MΦ) and more virulent in naïve mice than were non-host-adapted/MHB-grown bacteria (Loegering et al., 2006). Using *Ft* grown in BHI or MHB as models of host-adapted and non-host-adapted bacteria respectively, we found that *Ft* LVS and S4 grown in BHI produce/retain more capsular material. We found that this capsular material hinders various immune effectors and shortens the median survival time (MST) of naïve mice by 12–24 h (Hazlett et al., 2008; Zarrella et al., 2011). Faith et al. used an aerosol challenge model of naïve mice to assess the impact of growth in MHB, CDM, and BHI on the aerosolization and infectivity of *Ft* LVS. The authors found that growth in BHI was associated with better bacterial survival during aerosolization and a decreased LD_99_ (Faith et al., 2012). Collectively, the above findings indicate that *Ft* infection of naïve animals can be impacted modestly by the immediate growth history of the challenge agent. This is reminiscent of early work with another zoonotic adaptable pathogen, *Yersinia pestis* (*Yp*), in which inoculation of naïve mice with bacteria that were pre-adapted to mammalian environmental temperature resulted in a shorter MST (Rosenwald and Lincoln, 1966). This now is understood to result from adaptive changes in (i) the structure of *Yp* LPS (to a less inflammatory form) and (ii) regulated expression of virulence factors such as the capsular F1 Ag (Rebeil et al., 2004; Montminy et al., 2006).

While the impact of growth-media/adaptation status on *Ft* infection of naïve animals is developing, the role of these factors on tularemia vaccination is less well-explored. Recently, we reported that the immune-stimulatory nature of MHB-*Ft* makes inactivated forms of these bacteria better immunogens against challenge with *Ft* LVS (Kumar et al., 2016). Hypothetically, immunization outcomes could be also impacted by differential-growth of the challenge agent, which could display altered susceptibility to vaccine-induced immunity—at least until the challenge agent adapts to the host environment. Examples of this include subunit vaccines against zoonotic pathogens such as the Decorin-binding protein A (DbpA) of *Borrelia burgdorferi* (*Bb*) and the *Ft* outer membrane protein A (FopA). Immunization with DbpA protects mice against infection with *in vitro* cultivated *Bb* but not tick-transmitted *Bb*. This is due largely to the fact that DbpA is expressed *in vitro* but not by tick-borne spirochetes (Hagman et al., 2000; Blevins et al., 2008). Similarly, vaccination with purified FopA can provide significant protection against challenge with MHB-*Ft* LVS but not against challenge with either *Ft* S4 (Hickey et al., 2011) or BHI-*Ft* LVS (Hickey, 2011). The protective capacity of FopA immunization correlates with the lower levels of capsule and increased binding of FopA-specific Ab observed with MHB-*Ft* LVS (Hickey et al., 2011; Zarrella et al., 2011).

Here we used a battery of proteomic and serological analyses combined with immunization and challenge assays to further explore the impact of *Ft*'s phenotypic mutability on tularemia vaccine research. We tested the hypotheses that (i) *Ft* grown in BHI (BHI-*Ft*) displays a full protein composition more similar to that reported for infection-derived *Ft* and (ii) that this similarity would make BHI-*Ft* more susceptible to pre-existing, vaccine-induced immunity than MHB-*Ft*. Our findings indicate that, compared to *Ft* grown in MHB, BHI-*Ft* has increased proteomic similarity to host-adapted *Ft* and altered reactivity with infection-derived sera from multiple species. Significantly, in multiple experiments, with distinct immunization strategies, we found that immunized mice are significantly more susceptible to respiratory challenge with BHI-*Ft* S4 than to challenge with MHB-*Ft* S4. In effect, BHI-*Ft* S4 appears more virulent to immunized mice than does MHB-*Ft* S4 despite the fact that there is little difference in virulence to naïve mice.

## Materials and methods

### Bacteria and fractionation

The LVS strain of *Ft* was acquired from BEI (NR-646) and was grown *in vitro* in MHB, BHI, or BHI supplemented with casamino acids (BCA) as previously described (Hazlett et al., 2008; Hickey et al., 2011; Zarrella et al., 2011). LVS mutant strains used here have been described elsewhere (Su et al., 2007). *Ft* S4 was used within the CDC-certified A/BSL-3 facilities of Albany Medical College, the University of Pittsburgh, and the Trudeau Institute. Late-log phase *Ft* grown in MHB, BHI, or BCA were harvested by centrifugation (8,000 × g, 15 min, 20°C). Bacterial pellets were resuspended in the appropriate fresh growth media, transferred to pre-weighed Eppendorf tubes, pelleted as above after which the transfer supernatant was aspirated. Pellet wet weights were determined and used to estimate cell numbers based upon the estimate of 1 mg of wet weight = 5 × 10^8^ bacteria. Cells were resuspended to 2.5 × 10^7^/μl (higher bacterial concentrations promoted protein precipitation in subsequent steps) in 20 mM Tris, pH 8.0 containing 100 mM NaCl, 20 μl/ml protease inhibitor cocktail (Sigma, #P8849). After determining the protein concentrations (DC protein assay, BioRad, Hercules, CA), equilibrated bacterial suspensions were supplemented with EDTA to 5 mM, lysozyme to 100 μg/ml, Benzonase (1 μl/ml, Sigma), and Triton X-114 (Acros, NJ) to 1% from a 10% stock in PBS. Following a 1 h incubation at room temperature with periodic gentle agitation, a 1/20 vol. aliquot was saved as the whole-cell (WC) fraction; the WC and remaining sample were stored at −20°C overnight. The samples were thawed and incubated at RT until further viscosity reduction abated. Following 15 min incubation on ice, the samples were centrifuged (20 min, 16,500 × g, 4°C) to yield Triton X-114 soluble (TxS) and insoluble (TxI) fractions. The TxS fractions were transferred to fresh tubes and centrifuged again to remove any TxI carry-over; the TxI fractions were washed twice with cold PBS (1 vol). The TxS fractions were incubated in a 32°C water bath for ~10 min followed by centrifugation (7,000 × g, RT, 15 min) to effect phase partitioning (Bordier, 1981; Radolf et al., 1988; Sjostedt et al., 1991; Hazlett et al., 2001, 2005) into aqueous (top) and detergent (bottom) phases (any protein precipitation at this step rendered the samples useless, such experiments were discarded). Recovered phases were washed by supplementation (to 1% Triton X-114—A or 10 vol PBS—D) followed by phase separation; three washes per recovered phase were performed. The washed Triton insoluble (TxI) phases were resuspended in PBS containing 0.2% sarkosyl (Fisher Scientific, Fair Lawn, NJ) and incubated for 20 min with gentle agitation followed by centrifugation (30 min, 4°C, 16,500 × g). The sarkosyl-soluble (SS) fraction was recovered and centrifuged a second time to remove any carryover of insoluble material. The sarkosyl-insoluble (SI) material was washed once with PBS, 0.2% sarkosyl and dissolved in 10 mM Tris, pH = 8.0 containing 1% SDS. WC, A, D, SS, and SI fractions were resolved by SDS-PAGE. Occasionally the D, SS, and SI phases were pooled to represent the total membrane fraction.

### SDS-PAGE and western blot analysis

Samples derived from 10 μg of *Ft* protein (~1 × 10^8^ cells) were mixed with Laemmli sample buffer and boiled for 10 min prior to resolution through 4–12% gradient SDS-PAGE pre-cast gels (Invitrogen). The running buffer was NuPAGE MES SDS buffer from Invitrogen; gels were variously run at 90–160 V. Resolved gels were stained with either Coomassie blue (BioRad) or transferred to nitrocellulose membranes. Coomassie-stained gels were scanned into Adobe Photoshop using an HP 2820 scanner. Membranes were blocked for 30 min with PBS, 0.05% Tween 20, 5% non-fat dry milk. Primary Abs were applied for overnight incubation at dilutions ranging from 1:1,000 to 1:60,000. Blots to be probed multiple times were first probed with mAb prior to stripping and reprobing with polyclonal sera. HRP-conjugated secondary Abs were used at dilutions ranging from 1:1,000 to 1:20,000. Occasionally the secondary conjugate was a biotinylated goat α-species-specific Ab followed by a tertiary conjugate of streptavidin-linked HRP. Development of the chemiluminescent substrate (SuperSignal West Pico, Pierce, Rockford, IL) was visualized using an Alpha Innotech imaging system in movie mode. Densitometric analysis of developed blots was performed on the same system.

### Proteomics

#### Sample preparation for mass spectrometry

For each growth condition (MHB, BHI, or BCA), the WC and 4 subfractions (A, D, SS, SI) were run on SDS-PAGE gels either Full-Length (WC and A), or in a Gel Plug (D, SS, SI). Each fraction was mixed with sample buffer (w/β-ME) to a final vol of 100 μl. Full-Length: loaded 10 μl (1 × 10^8^ *Ft* eq)/lane on 4–12% SDS-PAGE gradient gels ran at 50 V for 4 h (Invitrogen). Gel-Plug: loaded 10 μl (5 × 10^8^ *Ft* eq)/lane on 12% SDS-PAGE gel ran at 50 V for 40 min to create “1D-Gel plug” (Invitrogen). All SDS-PAGE gels were stained with 25 mL Imperial Stain (Thermo 24615) for 1 h at 22°C and destained overnight at 4°C.

#### Trypsin digestion of samples from SDS-PAGE gels

*SDS-PAGE Gel Sectioning:* Samples from full-length SDS-PAGE gels (WC and A) were cut into 10 equal sections. Samples from the D1 and SS gel-plug SDS-PAGE were cut into two equal sections while the SI samples were cut into one section. *Trypsin*:breve:overDigestion: Protein bands to be analyzed, from SDS-PAGE gels, were excised by sterile razor blade and chopped into ~1 mm^3^ pieces. Each sample was washed in water and destained using 100 mM ammonium bicarbonate pH 7.5 in 50% acetonitrile. A reduction step was performed by addition of 100 μl 50 mM ammonium bicarbonate pH 7.5 and 10 μl of 10 mM Tris (2-carboxyethyl) phosphine HCl at 37°C for 30 min. Proteins were alkylated by adding 100 μl 50 mM iodoacetamide and allowed to react in the dark at 20°C for 30 min. Gel samples were washed in water, then acetonitrile, and dried in a SpeedVac. Trypsin digestion was carried out overnight at 37°C with 1:50 enzyme-protein ratio of sequencing grade-modified trypsin (Promega) in 50 mM ammonium bicarbonate pH 7.5, and 20 mM CaCl_2_. Peptides were extracted with 5% formic acid and vacuum dried.

#### Isotopic labeling

Peptide digests were reconstituted with 70 μl of Tris–HCl buffer solution (10 mM Tris-HCl, 150 mM NaCl, 20 mM CaCl_2_, pH 7.6), vortexed for at least 20 min to reconstitute the peptide mixture, then split into two vials of 30 μl each (^16^O vial and ^18^O vial), and 10 μl was retained unlabeled and stored in −80°C. In a separate vial, Mag-Trypsin beads (Clontech) were prepared as follows. Thirty microliters mag-trypsin beads per rxn (^16^O or ^18^O) were pooled and washed three times with 800 μl of Tris–HCl buffer solution, then brought back up in 30 ul/sample of Tris-HCl buffer, and aliquoted to a new 1.5 ml vial in the quantity of 30 μl, vortexing lightly after each aliquot to keep the mag-trypsin beads in suspension. Using a magnetic rack, the Tris-HCl buffer was removed from the beads, and the 30 μl of sample digest from above was added to the beads and vacuum dried. Thirty microliters of either ^16^O H2O or 97% ^18^O H_2_O (Cambridge Isotopes Laboratories) was added to the respective ^16^O or ^18^O prepared Mag-Trypsin bead vial and vortexed for 20 min to reconstitute the peptide mixture, and allowed to exchange overnight at 37°C. After ^18^O exchange, the solution was transferred to a new vial and any free trypsin in solution was inactivated with 1 mM PMSF for 30 min at 4°C. For each sample section, the digests were combined 1:1 as follows: Forward (FWD) Sample Set: (BHI)-^16^O:(BCA)-^18^O, or (BHI)-^16^O:(MHB)-^18^O. Reversed (REV) Sample Set: (BCA)-^16^O:(BHI)-^18^O, or (MHB)-^16^O:(BHI)-^18^O. Samples were dried and stored at −80°C until analysis. Three biological replicate experiments were performed.

#### HPLC for mass spectrometry

The peptide samples were loaded to a 0.25 μl C_8_ OptiPak trapping cartridge custom-packed with Michrom Magic C8 (Optimize Technologies), washed, then switched in-line with a 20 cm by 75 μm C_18_ packed spray tip nano column packed with Michrom Magic C18AQ, for a 2-step gradient. Mobile phase A was water/acetonitrile/formic acid (98/2/0.2) whereas mobile phase B was acetonitrile/isopropanol/water/formic acid (80/10/10/0.2). Using a flowrate of 350 nl/min, a 90 min, 2-step LC gradient was run from 5 to 50% B over 60 min, followed by 50–95% B over the next 10 min, hold 10 min at 95% B, back to starting conditions and re-equilibrated.

#### LC-MS/MS analysis

The samples were analyzed via electrospray tandem mass spectrometry (LC-MS/MS) on a Thermo LTQ Orbitrap XL, using a 60,000 RP survey scan, m/z 375–1,950, with lockmasses, followed by 10 LTQ CID scans on doubly and triply charged-only precursors between 375 and 1,500 Da. Ions selected for MS/MS were placed on an exclusion list for 60 s.

#### Database searching

*Spectral Count Samples*. Tandem mass spectra were extracted, charge state deconvoluted and deisotoped by MS-Convert.exe version 3.0.9490. All MS/MS samples were analyzed using Mascot (Matrix Science, London, UK; version 2.3.02) and X! Tandem [The GPM, thegpm.org; version CYCLONE (2010.12.01.1)]. Mascot and X! Tandem were set up to search uniprot_with_Isoforms_FRATH_07_2012.fasta (7/1/2012) assuming the digestion enzyme trypsin. Mascot and X! Tandem were searched with a fragment ion mass tolerance of 0.60 Da and a parent ion tolerance of 20 PPM. Oxidation of methionine, n-Formylation of the n-terminus and iodoacetamide derivative of cysteine were specified in Mascot and X! Tandem as variable modifications. ^18^*O Quant Samples*. All Isotopic ^*^.raw Data files were analyzed with MaxQuant version 1.2.2, searching against the uniprot_with_Isoforms_FRATH_07_2012.fasta database using the following criteria: ^18^O heavy label was selected for quantitation with a min of 1 high confidence peptide to assign quantitation H/L ratio. Trypsin was selected as the protease with max miss cleavage set to 2. Carbamiodomethyl (C) was selected as a fixed modification. Variable modifications were set to Oxidization (M), Formylation (*n*-term). Orbitrap mass spectrometer was selected using an MS error of 20 ppm and a MS/MS error of 0.5 Da. One percent FDR cutoff was selected for peptide, protein, and site identifications.

#### Criteria for protein identification

*Spectral Count Samples*. Scaffold (version Scaffold_4.5.1, Proteome Software Inc., Portland, OR) was used to validate MS/MS based peptide and protein identifications. Peptide identifications were accepted if they could be established at >95.0% probability by the Peptide Prophet algorithm (Keller et al., 2002) with Scaffold delta-mass correction. Protein identifications were accepted if they could be established at >99.0% probability and contained at least 1 identified peptide. Protein probabilities were assigned by the Protein Prophet algorithm (Nesvizhskii et al., 2003). Proteins that contained similar peptides and could not be differentiated based on MS/MS analysis alone were grouped to satisfy the principles of parsimony. ^18^*O Quant Samples*. Ratios were reported based on the MS level light and heavy peak areas determined by MaxQuant and reported in the proteinGroups.txt file as heavy/light. Proteins were removed from this results file if they were flagged by MaxQuant as “Contaminants,” “Reverse,” or “Only identified by site.” Complete three biological replicates were performed, with each biological replicate split into two technical replicates [^18^O forward (FWD) labeling, and ^18^O reverse (REV) labeling]. The abundance data from each biological replicate were normalized within Maxquant to the overall median peak intensity. Light and heavy peak intensities were analyzed in each run to determine protein hits that fell into the category of either (BHI-only hits or BCA-only hits) or (BHI-only hits or MHB-only hits) and retained if they confirmed to this state across all runs. In the case of (BHI-only or BCA-only protein hits) or (BHI-only or MHB-only protein hits), spectral counts can be used as a proxy for abundance.

#### Proteomics data analysis and presentation

*Spectral Counting*. For each biological replicate the total spectral counts derived from 1 × 10^8^ *Ft* (10 μg of protein) were used to calculate the mean (± standard deviation) spectral counts (SC) as a semi-quantitative proxy of protein abundance in each growth condition. In each growth condition the proteins were ranked by abundance with the protein having the highest mean spectral counts listed as number one. We also normalized abundance to a quasi-molar metric by dividing the mean spectral counts by 10 for each 10 kDa of mass for each protein; this was used in recognition of the fact that large proteins generate more tryptic peptides, and therefore spectral counts, than smaller proteins. ^18^*O labeled-peptides*. For each biological replicate tryptic peptides were subject to duplicate technical analysis with reciprocal-labeling to determine the mean FC for each biological replicate. The data reported here is the mean FC and standard deviation derived from the means of two to three biological replicates. To determine which mean FCs were statistically significant we performed a *t*-test of the mean FC compared to a FC = 1 (no change) and *p* > 0.05 were considered significant.

### Infection-derived immune sera used for western blotting

*S4-convalescent mouse serum*. Six to eight week old C57BL/6 mice (Taconic, Germantown, NY) of either sex (*n* = 10) were used for raising antisera against *Ft* S4. Mice were anesthetized by intraperitoneal injection of a cocktail of Ketamine (5 mg/kg) (Fort Dodge Animal Health, Fort Dodge, IA) and Xylazine (4 mg/kg) (Phoenix Scientific, St. Joseph) and checked for the loss of toe-pinch reflex. Anesthetized mice were immunized intranasally (i.n.) on day zero with 2 × 10^3^ colony forming units (CFU) of live, MHB-grown *Ft* LVS in 20 μl (10 μl/nare) of sterile PBS. Identical booster immunizations were administered on d14 and d28. On d42, the immunized mice were challenged i.n. with 26 CFU of MHB-grown *Ft* S4. One hundred percent of these mice survived. The same mice were challenged again with 100 CFU of MHB-grown *Ft* S4 on d72; again all mice survived. On d100, mice were re-challenged with 500 CFU of MHB-grown *Ft* S4. On day 111, the surviving mice (100%) were bled by retro orbital venipuncture; sera were harvested and stored at −80°C until further use. The immunization and challenge doses were confirmed by plating serial dilutions on MHB chocolate agar plates. The plates were incubated at 37°C in the presence of 5% CO_2_ for 48 h and the colonies were counted. *S4-convalescent rabbit sera*. Rabbit sera used here was previously generated (Reed et al., 2014) in New Zealand White rabbits by scarification on day zero with BHI-grown *aroD Ft* S4 followed by aerosol challenge on d28 with 50–500 LD_50_ of BHI-grown WT *Ft* S4. Sera were harvested on d56. *LVS-immunized, human sera*. The USAMRIID Special Immunization Program supplied aliquots of previously-banked, pre- and post- LVS-immunization sera from 10 sero-positive, de-identified study participants. Provision and use of these human sera as described herein was approved by the Institutional Review Boards of USAMRIID and Albany Medical College. The Institutional Animal Care and Use Committees (IACUCs) of Albany Medical College, the University of Pittsburgh, and the Trudeau Institute approved animal protocols used herein.

### Murine vaccination and challenge

Four independent challenge experiments were conducted. In the first two, conducted at Albany Medical College and the University of Pittsburgh respectively and shown in **Figure 4**, groups of female BALB/c mice (*n* = 6–8/group) were immunized three times on d0, d14, and d28 with PBS or 200 CFU of live *Ft* LVS. Mice were challenged on d42 with *Ft* S4 and monitored for 14d. For the AMC trial, mice (Taconic) were primed intradermally (i.d.) and boosted twice intranasally (i.n.) with PBS or live, MHB-*Ft* LVS prior to i.n. challenge with 25 CFU of MHB-*Ft* S4. For the University of Pittsburgh trial, mice (Charles River) were primed i.n. and boosted twice i.n. with PBS or BHI- *Ft* LVS prior to challenge with 34 CFU of BHI-*Ft* S4 delivered via aerosol. In the third experiment (**Figure 5**), two groups (*n* = 20/group) of C57/BL6 mice (Taconic; ½ male, ½ female) were primed i.d. and twice boosted i.n. on d11 and d25 at AMC with either PBS or 200 CFU of live, MHB-*Ft* LVS. Mice were challenged i.n. on d42 at the Trudeau Institute with 26 ± 2 CFU of either MHB- or BHI-*Ft* S4 and monitored for 21 d. In our final experiment (**Figure 6**), two groups (*n* = 22–24/group) of C57/BL6 mice (Taconic; ½ male, ½ female) were primed i.d. and boosted i.n. once on d21 at AMC with either PBS or 1,000 CFU of live, BHI-*Ft* LVS. On d42 the mice were challenged i.n. with 29.5 ± 2.5 CFU of *Ft* S4; half of each group was challenged at AMC with MHB-*Ft* S4; the other half was challenged at the Trudeau Institute with BHI-*Ft* S4. Survival was monitored for 21 d. The IACUCs of Albany Medical College, the University of Pittsburgh, and the Trudeau Institute approved animal protocols used herein. All challenge-survival data are presented as Kaplan–Meier survival curves with statistical analysis by the Log-rank (Mantel–Cox) test.

## Results

Previously, in an effort to identify *in vitro* cultivation conditions for *Ft* that yielded “host-adapted”-like bacteria, we used a variety of biochemical and immunological approaches to compare *Ft* grown in MHB and BHI to *Ft* grown within macrophages (MΦs). Collectively our findings, based on ~10 bacterial and/or immunological parameters, suggested that BHI-*Ft* display a phenotype similar to that of infection-derived *Ft* whereas the phenotype of MHB-*Ft* is distinct (Hazlett et al., 2008; Zarrella et al., 2011; Singh et al., 2013). To extend our understanding of these phenotypes, here we performed a comprehensive proteomics analysis of *Ft* LVS grown to late-log phase in broths of MHB and BHI [without and with supplementation of casamino acids (BCA)]. BCA was included here as we previously found that the addition of casamino acids to BHI down-regulates several Igl proteins (Hazlett et al., 2008; Zarrella et al., 2011). We acquired two types of LC-MS/MS data. The first was label-free, spectral counting which provides a semi-quantitative measurement of a protein's abundance relative to other proteins in the same sample. We also used ^18/16^O_2_ labeling which provides a highly accurate fold change (FC) of a protein's abundance between two distinct samples. A comprehensive tally of our proteomics data, alongside the published findings of others, is presented in Table S1.

### Overview and comparison with existing datasets

We detected expression of 1,156 distinct *Ft* LVS proteins, 916 in the ^18/16^O_2_ labeled samples and an additional 240 by label-free detection. Our total detection rate of 67% of the hypothetical *Ft* proteome is identical to that reported for a related quantitative proteomics strategy recently used by Lenco et al. to interrogate the proteomes of *Ft* LVS and *Ft* S4 (Lenco et al., 2009). Of the 845 proteins for which we have high-quality quantitative data, 17% were more abundant [>1.5 FC that was statistically significant] in BHI-*Ft* whereas 9% were less abundant (Table S1).

**Figure 1:**
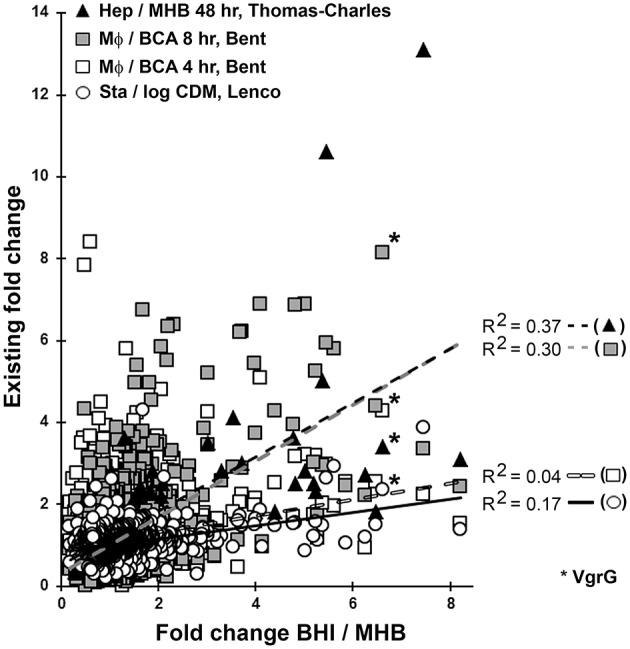
Comparative omics. The fold changes (FC) for each of the 845 proteins we quantified in both BHI- and MHB-grown *Ft* were plotted against the transcriptional FC for the same genes determined by Bent et al. (2013) at 4 h (white squares), 8 h (gray squares), and Thomas-Charles et al. (2013) (black triangles). Four lines of best fit (least squares, best fit linear regression) are shown for comparison of our data with that of Bent 4 h (*R*^2^ = 0.04), Bent 8 h (*R*^2^ = 0.30), Thomas-Charlie (*R*^2^ = 0.37), and Lenco et al. (2009) (*R*^2^ = 0.17, open circles solid black line). Values for VgrG (6.6—BHI/MHB; 8.1—Bent, 8 h; 4.3—Bent, 4 h; 3.4—Thomas Charles, 48 h; 2.4—Lenco) are indicated by asterisks to facilitate interpretation of the data. MHB, Mueller-Hinton Broth; BHI, Brain Heart Infusion Broth; MΦ, Macrophages; BCA, BHI supplemented with Casamino Acids; CDM, Chamberlain's Defined Medium; Stat., Stationary Phase; Log, Logarithmic phase.

As the quality of our *Ft* data appeared to be comparable to that of others, we next compared our BHI/MHB proteomics FCs to existing “omics” FC datasets in which *Ft* grown in mammalian cells and/or mice were compared to bacteria grown in broth (Twine et al., 2006a; Wehrly et al., 2009; Bent et al., 2013; Thomas-Charles et al., 2013). This comparison was possible as FC is a form of data expression common to both “omics” technics. Our goal was to very-broadly test the notion that BHI-*Ft* is more similar, than MHB-*Ft*, to what has been reported for host-adapted *Ft*. While comparing FCs across distinct “omics” platforms and between different laboratories presents some limitations, we perused this analysis with an eye toward broad trends (operons, regulons, and protein families) that were apparent in our data and within multiple reports for host-adapted *Ft*. As a control, we also included the proteomics data of Lenco et al. who analyzed *Ft* grown *in vitro* to stationary and log phase in CDM (Lenco et al., 2009). Our BHI/MHB FC dataset best fit the transcriptional datasets of Bent et al. (FCs derived from *Ft* L Daygrown in MΦs for 4 and 8 h vs. in BCA) (Bent et al., 2013) and Thomas-Charles et al. (FCs derived from *Ft* LVS grown in hepatocytes for 2 d vs. in MHB) (Thomas-Charles et al., 2013). A linear regression analysis of our data against the Bent et al. 4 h dataset revealed a very low correlation (*R*^2^ = 0.04, Figure 1); the correlation between our data and that of Lenco was moderately increased (*R*^2^ = 0.17). Our data showed the highest correlation with data derived from 8 and 48 h of intracellular growth (*R*^2^ = 0.30 and 0.37 respectively); these latter two *R*^2^-values were not significantly different from each other (correlation significance analysis, http://vassarstats.net/). Collectively, these results suggest that on a broad scale, BHI-*Ft* more closely reflect what has been reported for bacteria that have grown in host cells for 8–48 h.

### Virulence factors

Identifying the virulence factors (VF) of *Ft* has been a significant area of interest for the tularemia field. Through the use of mutant *Ft* and various infection models, a collection of the bacterium's VFs is emerging (Baron and Nano, 1998; Santic et al., 2005, 2007; Brotcke et al., 2006; Tempel et al., 2006; Su et al., 2007; Bonquist et al., 2008; Sammons-Jackson et al., 2008; Barker et al., 2009; Buchan et al., 2009; Dean et al., 2009; Schmerk et al., 2009; Ahlund et al., 2010; McCaffrey et al., 2010; Broms et al., 2011, 2012a; Straskova et al., 2012; Robertson et al., 2013, 2014) and currently contains 190 *Francisella* proteins. In our proteomics analysis, we detected 92% of these proteins which is significantly higher (*p* < 0.0002; proportion analysis http://vassarstats.net/) than our overall *Ft* protein detection rate (67%). Among these VFs, 26% were up-regulated by 1.5-fold or more in BHI-*Ft* compared to MHB-*Ft*; 9% were down-regulated in BHI (Table S1). The percentage of BHI up-regulated VFs was significantly higher (*p* = 0.013; proportion analysis http://vassarstats.net/) than the percentage of BHI up-regulated proteins in the broader proteome (17%).

The most studied VFs of *Ft* include the 17 *Francisella* Pathogenecity Island (FPI) proteins, which likely form a type-6 secretion system for the delivery of *Ft* effector proteins into target cells (de Bruin et al., 2007; Ludu et al., 2008; Broms et al., 2009, 2012a,b; Clemens et al., 2015). As shown in Table 1 (and Table S1), we detected all FPI proteins with the exception of the 135 kDa protein PdpD (FTT1715)—whose gene is truncated in *Ft* LVS (Lenco et al., 2009). The average FC for the FPI proteins (excluding IglF [high level of variation]) was 5.3 in BHI-*Ft* relative to MHB-*Ft*. In general, our FPI data compares favorably with that of Bent et al. and Thomas-Charles et al. particularly for the later time points of intracellular (IC) growth (Table 1 and Table S1). Similarly, our findings generally mirror those derived from mouse and MΦ models with *Ft* S4. Twine et al. purified *Ft* S4 from murine spleens 4 d post-infection and used proteomics to compare these to MHB-*Ft* (Twine et al., 2006a); Wehrley et al. used transcriptional analysis to compare *Ft* S4 grown within cultured MΦs (for 1–24 h) to *Ft* S4 grown in CDM (Wehrly et al., 2009). Based on the available *Ft* S4 data, the FPI expression we observed with *Ft* LVS in BHI appears to echo S4 FPI induction reported in *Ft* from mouse spleens (Twine et al., 2006a) and in MΦs at ~12–16 h (Wehrly et al., 2009) (Table S1). To confirm key findings of our proteomics data, we performed SDS-PAGE and western blot analysis of MHB-, BHI-, and BCA-grown *Ft* LVS along with BHI-grown *Ft* S4. As shown in Figure S1, BHI-grown *Ft* contained elevated levels of VgrG (FTL0123/FTT1702), IglC (FTL0113/FTT1712), and IglB (FTL0112/FTT1713). IglC and IglB levels were comparable in BHI-grown LVS and S4 while VgrG levels were slightly higher in S4 than LVS—as previously reported (Lenco et al., 2009).

**Table 1:** A sub-set of our proteomics data.

**Locus (LVS, S4)**	**Protein (MW-kDa)**	**Spectral counts**		**Fold changes**			**VF**	**FevR regulon**	**Times reports as an antigen**
		**% Sol**.	**Abundance Rank MHB, BHI**	**BHI/MHB**	**BHI/BCA**	**MΦ/BCA, Bent, 4 h, 8 h**			
1,191, 1,269	DnaK (69)	93	3, 3	**0.7** ± 0.1	1.2 ± 0.1	1.0, 0.9	Y		7
1,714, 1,696	GroE (57)	96	1, 1	**1.2** ± 0.0	**1.2** ± 0.0	0.5, 0.6	Y		8
1,751, 137	EFTu (43)	92	4, 4	0.8 ± 0.2	**0.6** ± 0.1	1.0, 0.5			5
1,328, 583	FopA (41)	16	31, 59	0.8 ± 0.2	0.8 ± 0.1	**1.7, 1.5**	Y		6
**FPI PROTEINS**									
126, 1,699	PdpA (95)	92	263, 24	**4.8** ± 1.1	**1.8** ± 0.1	**3.2, 6.9**	Y	Y	0
125, 1,700	PdpB (125)	35	175, 26	6.6 ± 5.4	**1.9** ± 0.0	**1.9, 4.9**	Y		0
124, 1,701	IglE (14)	0	955, 772	3.7	2.7 ± 1.3	2.0, **6.2**	Y		0
123, 1,702	VgrG (18)	99	546, 224	6.6 ± 2.1	2.3 ± 1.0	**4.3, 8.1**	Y		0
122, 1,703	IglF (65)	80	868, 435	11.9 ± 13	**0.1** ± 0.2	2.4, **6.3**	Y	Y	0
121, 1,704	IglG (18)	–	ND, 910	ND	0.7 ± 1.0	**3.0, 6.1**	Y		0
120, 1,705	IglH (55)	91	400, 95	6.5 ± 3.6	1.9 ± 0.9	**2.5, 4.4**	Y	Y	0
119, 1,706	DotU (25)	61	487, 308	3.7 ± 2.3	1.0 ± 0.9	2.2, **2.9**	Y	Y	0
118, 1,707	IglI (45)	81	305, 57	5.4 ± 2.1	**2.8** ± 0.5	**2.0, 3.0**	Y	Y	0
117, 1,708	IglJ (30)	87	914, 501	5.2 ± 2.4	**2.3** ± 0.4	1.7, **3.0**	Y	Y	1
116, 1,709	PdpC (156)	80	117, 7	6.3 ± 3.9	**2.8** ± 0.3	0.9, **2.2**	Y	Y	0
115, 1,710	Hcp (22)	82	971, 489	8.2 ± 3.9	2.0 ± 0.6	1.5, **2.4**			0
114, 1,711	IglD (46)	90	328, 117	**4.8** ± 0.8	**1.3** ± 0.0	1.7, **4.0**	Y	Y	0
113, 1,712	IglC (23)	96	59, 19	**4.0** ± 0.4	4.0 ± 1.8	**2.0, 5.4**	Y	Y	2
112, 1,713	IglB (55)	82	23, 6	**4.0** ± 1.0	1.7 ± 0.2	**2.1, 3.7**	Y	Y	2
111, 1,714	IglA (21)	83	310, 61	4.4 ± 1.4	1.8 ± 0.3	**2.5, 4.3**	Y	Y	0
NA, 1,715	PdpD, NA	ND	ND	ND	ND	NA	Y		0
FPI protein average^*^				5.3	2.2	2.2, 4.6			
**KNOWN FPI REGULATORS**									
449, 383	FevR (13)	83	951, 655	**5.0** ± 0.3	2.6 ± 1.8	**3.2, 6.9**	Y	Y	0
552, 1,557	PmrA (26)	99	144, 175	2.0 ± 0.9	**0.8** ± 0.1	0.6, 0.8	Y	Y	0
1,606, 458	SspA (24)	92	417, 399	0.8 ± 0.2	1.3 ± 0.1	0.6, 1.2	Y		0
1,185, 1,275	MglA (22)	100	522, 654	0.6 ± 0.3	0.9 ± 0.1	1.9, 0.4	Y		0
1,184, 1,276	MglB (15)	-	836, 963	ND	ND	1.6, 0.8	Y		0
**NON-FPI MARKERS of GROWTH HISTORY**									
1,503, 720	Dgt (50)	100	599, 360	**5.2** ± 0.3	**1.7** ± 0.1	**3.2, 5.3**			0
673, 1,390	PanC (30)	95	492, 225	**4.1** ± 0.7	1.4 ± 0.3	1.0, 1.1		Y	0
675, 1,388	PanG (27)	88	743, 317	**3.5** ± 0.7	0.7 + 0.4	1.8, 2.9		Y	0
207, 296	Pcp (24)	91	315, 178	3.0 ± 1.1	**1.7** ± 0.1	**4.3, 5.2**	Y	Y	0
1,832, 29	FslA (74)	74	643, 295	5.5 ± 4.8	2.4 ± 0.3	**3.0, 5.9**	Y	Y	0
1,834, 27	FslC (47)	89	856, 333	7.5 ± 3.1	0.9 ± 0.2	2.2, 3.4		Y	0
78, 1671	RibD (40)	100	449, 792	**0.43** ± 0.0	**0.11** ± 0.0	1.1, 1.0	**<s/>**		0
1,739, 149	MetK (42)	91	212, 542	**0.35** ± 0.0	0.54 ± 0.2	0.6, 0.5			0
1,478, 1,317	GuaB (52)	100	12, 55	**0.35** ± 0.0	**0.37** ± 0.2	**0.3**, 0.7	Y		1
**LPS/CAPSULE ENZYMES**									
606, 1,450	WbtM (39)	100	334, 300	**2.1** ± 0.3	0.7 ± 0.1	0.9, 1.6	Y	Y	0
600, 1,456	WbtH (72)	67	247, 242	**1.7** ± 0.0	0.9 ± 0.3	**0.3, 0.3**			0
596, 1,460	WbtE (49)	98	74, 64	**1.5** ± 0.1	1.0 ± 0.2	**0.5, 0.5**	Y		0
595, 1,461	WbtD (42)	94	476, 402	1.5 ± 0.2	0.6 ± 0.3	**0.5, 0.5**	Y	Y	0
1,399, 1,478	KdsB (28)	93	276, 238	1.8 ± 0.4	1.0 ± 0.0	1.2, **2.2**			0
609, 1,447	ManB (55)	100	558, 585	**1.6** ± 0.2	0.6 ± 0.6	0.8, 0.9	Y		0
**RIBOSOMAL PROTEINS**									
47 ribosomal proteins for which we have matched data				**24** down	**8** down	**12** down, **32** down			

FTL (LVS) and FTT (S4) locus numbers are indicated along with the proteins name and mass predicted from the LVS genome. Data based upon label-free spectral counts include solubility [the percentage of each protein's total tryptic peptides detected in either the soluble (S) or membrane (M) fractions] and abundance rank for each protein in MHB-Ft and BHI-Ft. Fold change data is based on reciprocal ^16^O and ^18^O labeling performed in biological duplicate to yield the indicated mean and standard deviations. Numerical data in bold font represent statistically significant changes [for each protein the status as a virulence factor (VF) was drawn from (Baron and Nano, 1998; Lai et al., 2001; Santic et al., 2005; Brotcke et al., 2006; Maier et al., 2006; Tempel et al., 2006; Maier et al., 2007; Santic et al., 2007; Su et al., 2007; Thomas et al., 2007; Bonquist et al., 2008; Sammons-Jackson et al., 2008; Barker et al., 2009; Buchan et al., 2009; Dean et al., 2009; Schmerk et al., 2009; Ahlund et al., 2010; McCaffrey et al., 2010; Broms et al., 2011, 2012a; Straskova et al., 2012; Robertson et al., 2013, 2014; Rasmussen et al., 2015)]; status as a FevR regulon member was drawn from Charity et al. (2007, 2009), Brotcke and Monack (2008), and Ramsey et al. (2015); the number of times each protein has been reported as a target of infection-derived Ab was drawn from references (Twine et al., 2006b, 2010, 2012a; Janovska et al., 2007; Sundaresh et al., 2007; Savitt et al., 2009; Fulton et al., 2011; Nuti et al., 2011; Golovliov et al., 2013; Chu et al., 2014), and the transcriptional fold changes upon intracellular growth for 4 and 8 h were drawn from Bent et al. (2013).

* *Excludes values for IglF*.

Regulation of *Ft* virulence is also an active area of investigation with ~15 *Ft* proteins identified as having various degrees of influence on VF/FPI gene expression. The most well-characterized of these are MglA (FTL1185) and SspA (FTL1606) (homologs of the *E. coli* stringent starvation protein A—SspA), and the DNA-binding protein FevR (FTL0449, also called PigR). In the presence of ppGpp, FevR binds both to DNA up-stream of VF promoters and to a protein complex including MglA, SspA, and RNA polymerase to affect transcription of the MglA/SspA/FevR regulon which contains many *Ft* VF genes—including *fevR* and most of the (Brotcke et al., 2006; Charity et al., 2007, 2009; Brotcke and Monack, 2008; Rohlfing and Dove, 2014; Ramsey et al., 2015). We observed a 5.0 FC for FevR in BHI-*Ft* relative to MHB-*Ft* (Table 1 and Table S1)—values that closely track the average FPI FC. Among the remaining FPI regulators, we observed that PmrA (FTL0552) levels were slightly higher (FC = 2.0, Table 1 and Table S1) in BHI-*Ft* as we previously reported (Hazlett et al., 2008). Levels of SspA, OxyR (FTL1014), QscC (FTL1762), TrmE (FTL1177), SpoT (FTL1413), Fur (FTL1831), and Hfq (FTL0898) were unchanged, and levels of MglA, MglB (FTL1184), MigR (FTL1542), and CphA (FTL0831) trended lower in BHI-*Ft* (Table 1 and Table S1). The changes we observed for the FPI regulators (particularly for FevR) in BHI-*Ft* appear to mirror those reported for bacteria grown in host cells (Wehrly et al., 2009; Bent et al., 2013) and are greater than that observed for *Ft* grown to stationary phase in CDM (Lenco et al., 2009).

Generation of ppGpp to activate the FevR regulon is dependent on RelA (FTL-0285) and/or SpoT activity (Charity et al., 2009) and is inducible through amino acid starvation experimentally-triggered by serine hydroxamate (Dean et al., 2009; Faron et al., 2013). Under these conditions, ppGpp levels rise by 2- to 3-fold and correspond with a 2- to 3-fold increase in *iglA* promoter activity (Faron et al., 2013). By comparing the proteomes of *Ft* grown in BHI and BCA [BHI supplemented with casamino acids (the amino acid source in MHB)] we were able to explore the extent to which amino acid starvation contributes the differences in MHB- and BHI-grown *Ft*. Consistent with the reported impact of serine hydroxamate, we found the absence of casamino acids induced an average FPI protein induction of 2.2-fold and a 2.6 induction of FevR (Table 1). These values are similar to those reported for *Ft* grown in MΦ for 4 h (ave FPI-2.2, FevR 3.2) but lower than the full FPI induction reported for *Ft* grown in MΦ for 8 h (ave-FPI 4.6, FevR 6.9) and what we observed between BHI- and MHB- grown *Ft* (ave FPI-5.3, FevR 5.0). These data suggest that while amino acid limitation alone can induce the FevR regulon 2- to 3-fold, additional, possibly nutritional, signals contribute to full FPI induction. Classically, nutrient starvation and induction of the stringent starvation response (mediated by RelA/SpoT and ppGpp) results in decreased abundance of ribosomes. Of the 47 ribosomal proteins for which we had robust FC data, 24 were statistically less abundant in BHI-*Ft* compared to MHB-*Ft* (Table 1 and Table S2) Among these 47 proteins, 8 were reduced in BHI compared to BCA; 12 and 32 are reportedly reduced after 4 and 8 h respectively of growth in MΦ. Thus in our data, as well as that of Bent for LVS (Bent et al., 2013) and Lenco for LVS and S4 (Lenco et al., 2009), FPI induction appears to correlate with ribosomal protein reduction.

Collectively, the protein fingerprint of log-phase BHI-*Ft* (compared to MHB *Ft*) is one of nutrient-depleted bacteria that—through induction of a stringent starvation-like response—have induced the FevR regulon for high-level expression of the bacterium's VFs.

### LPS and capsule synthesis

Another category of *Ft* proteins that contribute significantly to virulence are the ~65 enzymes that synthesize/modify/incorporate carbohydrates into the bacterium's surface-coating of polysaccharides. Principal among these polysaccharides is the four sugar, O-antigen moiety synthesized by the Wbt enzymes and incorporated into polymers found in both the bacterium's LPS and the discrete O-Ag capsule. Previously, we demonstrated that BHI-*Ft* have higher molecular weight capsular polysaccharides and longer polymers of LPS O-Ag compared to MHB-*Ft* although the mechanistic basis for this observation was unknown (Zarrella et al., 2011). In our proteomics data (Table 1 and Table S1) for BHI-*Ft*, we observe moderately increased levels of several Wbt enzymes (M, H, E, and D) as well as KdsB and ManB—enzymes that contribute to synthesis of the KDO core which links O-Ag to Lipid A. Among the remaining ~40 LPS/Capsule related proteins (Lpx, Waa, Lpc, Lpt, Flm, and Nax members) for which we have robust data, no significant differences were noted between MHB-*Ft* and BHI-*Ft* (Table S1). As with many *Ft* LPS/capsule synthases, perturbations of WbtM, WbtE, WbtD, and ManB reduce the bacterium's polysaccharide coat rendering the pathogen highly susceptible to host defenses (Thomas et al., 2007; Lai et al., 2010; Lindemann et al., 2011; Twine et al., 2012b; Okan et al., 2013; Rasmussen et al., 2015). Currently, it is unclear if the increased abundance of the Wbt, Kds, and Man enzymes reported here for BHI-*Ft* is sufficient to account for the increased surface polysaccharides we previously described. Another potential factor, increased retention of capsular material, has also been postulated for BHI-*Ft* (Faith et al., 2012) and a mechanism of such retention, lipid-modification of O-Ag capsule, has recently been reported for BHI-*Ft* (Barker et al., 2016).

### Immuno-dominant antigens

**Figure 2:**
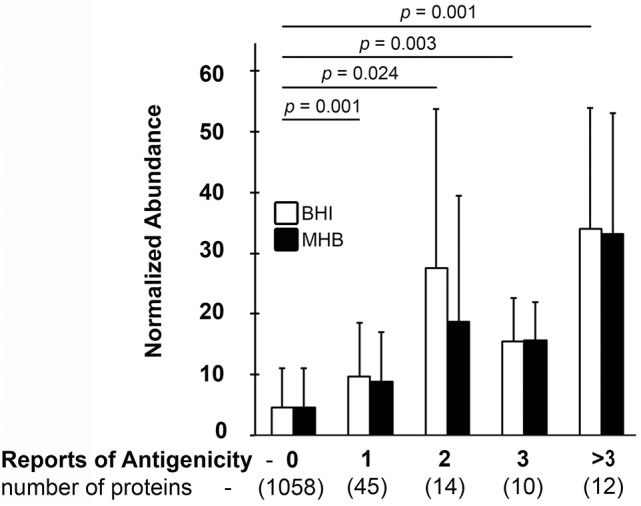
*Ft* protein abundance correlates with antibody reactivity. The average normalized abundance of the 1,139 *Ft* proteins we detected (Y-axis) was plotted against the number of times (0 to >3) that each protein has been reported as Ab reactive with infection-derived sera (X-axis). X axis values in parentheses indicate the number of *Ft* proteins with the indicated number of reports of sero-reactivity (0 to >3). Means and standard deviations from two to three biological replicates are indicated; statistical analysis was via 2-tailed, heteroscedastic *T*-test with Bonferroni correction with four comparisons. Indicated *p*-values are for the BHI dataset.

To date, 81 *Francisella* proteins have been reported one or more times to be immunogenic in an immunization or sub-lethal infection model (Twine et al., 2006b, 2010, 2012a; Janovska et al., 2007; Sundaresh et al., 2007; Savitt et al., 2009; Fulton et al., 2011; Nuti et al., 2011; Golovliov et al., 2013; Chu et al., 2014). We detected 100% of these proteins, which is significantly (*p* < 0.0002) higher than our overall *Ft* protein detection rate (67%). Among the immunogenic proteins, 18% were more abundant in BHI-*Ft* while 13% were more abundant in MHB-*Ft*. In the course of our analysis we made an intriguing observation—the proteins most frequently reported to be immunogenic were among those that we measured as the most abundant. For example, in our label-free, spectral count data, GroEL (FTL1714) and DnaK (FTL1194) were the 1st and 3rd most abundant proteins in *Ft* (Table 1) and these proteins have been described as immunogenic by nearly every group that has performed *Ft* immunoproteomics. To further explore the relationship between protein abundance and reports of immunogenicity, we plotted the spectral counting abundance (normalized for protein size as larger proteins generate more peptides) for >1,100 proteins against the number of published reports of immunogenicity for each protein. As shown in Figure 2 there is a strong linear trend between a protein's abundance and the likelihood that the protein will have been reported as immunogenic. This trend was similar whether we used data from BHI- or MHB-grown *Ft*. The mean normalized abundance for the proteins in each category (1 report up to >3 reports) was significantly higher than the abundance of proteins that had not been reported as immunogenic.

**Figure 3:**
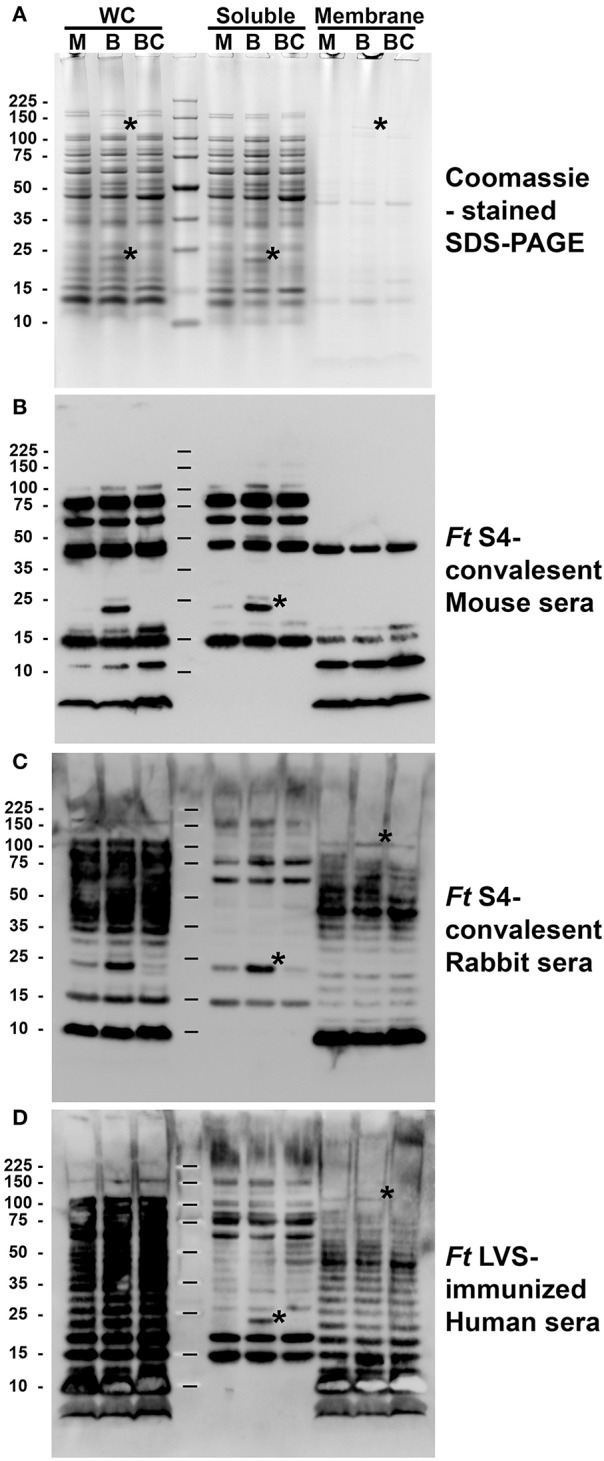
Differentially expressed *Ft* proteins are immunogenic in multiple models of infection. Whole cells (WC) lysates of *Ft* LVS grown in MHB (M), BHI (B), or BCA (BC) were partitioned into soluble and membrane fractions prior to SDS-PAGE **(A)** and western blot analysis **(B–D)**. Each lane was loaded with material derived from 1^*^10e8 *Ft*. Asterisks in **(A)** indicate 2 examples of coomassie-visable, differentially-abundant proteins, one soluble ~23 kDa and one ~120 kDa membrane. Replicate blots were independently probed with sera from S4-convalescent, inbred (C57/BL/6) mice; a S4-convalescent, outbred rabbit; and an LVS-vaccinated human. Asterisks in **(B–D)** indicate two examples of differentially-detected moieties, one soluble ~23 kDa and one ~120 kDa membrane.

As several immunogenic proteins are also differentially expressed to become highly abundant in BHI-*Ft* (Table 1), we hypothesized that sera from S4-surviving subjects might react differently with *Ft* that had been differentially cultivated. Inspired by recent reports from the Bosio group (Griffin et al., 2015; Roberts et al., 2016) indicating that C57BL/6 mice can be successfully immunized against challenge with MHB-*Ft* S4, we generated S4-convalescent mouse sera using live LVS in a prime-boost-boost strategy followed by an increasing challenge-dose regimen (26-100-500 CFU) with MHB-*Ft* S4. As a source of outbred S4-convalescent Ab, we used rabbits that had been primed with *Ft* S4 Δ*aroD* and challenged with BHI-*Ft* S4 prior to serum collection (Reed et al., 2014). For comparison, we also used sera from another outbred population, LVS-immunized humans. With these 3 sera, we probed by western blot, whole cell (WC), soluble, and membrane fractions of *Ft* LVS that had been grown in MHB, BHI, or BCA. As shown in Figure 3, the outbred rabbit and human sera strongly recognized the typical “ladder pattern” indicative of reactivity with the LPS O-antigen (Ag). The ladder pattern was present in the WC and membrane fractions of WT *Ft* LVS (Figure 3) and absent in preparations from O-Ag mutant *Ft* (data not shown). In contrast, our hyper-immune Mo sera displayed markedly reduced LPS-reactivity and instead showed prominent reactivity with a discrete number of soluble and membrane proteins (Figure 3); this pattern was unchanged when we probed preparations from O-Ag deficient *Ft* (data not shown). A similar discord between human and mouse immune serum reactivity with *Ft* LPS has been noted previously by Huntley et al. (2007).

Among the protein Ags recognized by these sera were several differentially abundant species. In particular, all three sera reacted with a soluble ~ 23 kDa moiety present in BHI-*Ft* at apparently higher levels than in MHB-*Ft* or BCA-*Ft* (Figures 3B–D, soluble, asterisk). This pattern of Ab-reactivity was mirrored by the abundance of a ~23 kDa soluble protein visible on coomassie-stained SDS-PAGE gels (Figure 3A) and by the reactivity with mAb against IglC (Figure S2). Within the membrane fractions, the only visible, differentially abundant Ag was a ~120 kDa band of reactivity in the outbred Rb and Hu sera (Figures 3C,D, membrane, asterisks). The abundant O-Ag reactivity of these sera may obscure additional differentially abundant membrane species. Collectively, these results with sera from multiple models and immunization schedules indicate that vaccine-induced immunity recognizes several differentially-expressed *Ft* Ags.

### Differential growth of *Ft* S4 governs the efficacy of prophylactic immunization

As several Ag differences in MHB-*Ft* and BHI-*Ft* were recognized by immune/convalescent sera, we hypothesized that immunized hosts might be differentially susceptible to challenge with these two forms of the bacteria. Previously, we observed that naïve mice infected with BHI-*Ft* (LVS or S4) showed a slightly shorter median survival time (MST) compared to that of mice challenged with MHB-*Ft* (Hazlett et al., 2008; Zarrella et al., 2011). Here, we envisioned two distinct scenarios for immunized mice challenged with *Ft* S4 previously grown in MHB or BHI. The elevated reactivity of the differentially-expressed, dominant Ags could render a challenge inoculum of BHI-*Ft* S4 more susceptible to pre-existing immunity (which could result in increased host survival). Alternatively, the thicker carbohydrate coating, coupled to the immediate benefit of pre-formed VFs, could make the BHI-*Ft* S4 challenge inoculum less susceptible to pre-existing immunity (which should result in decreased host survival).

**Figure 4:**
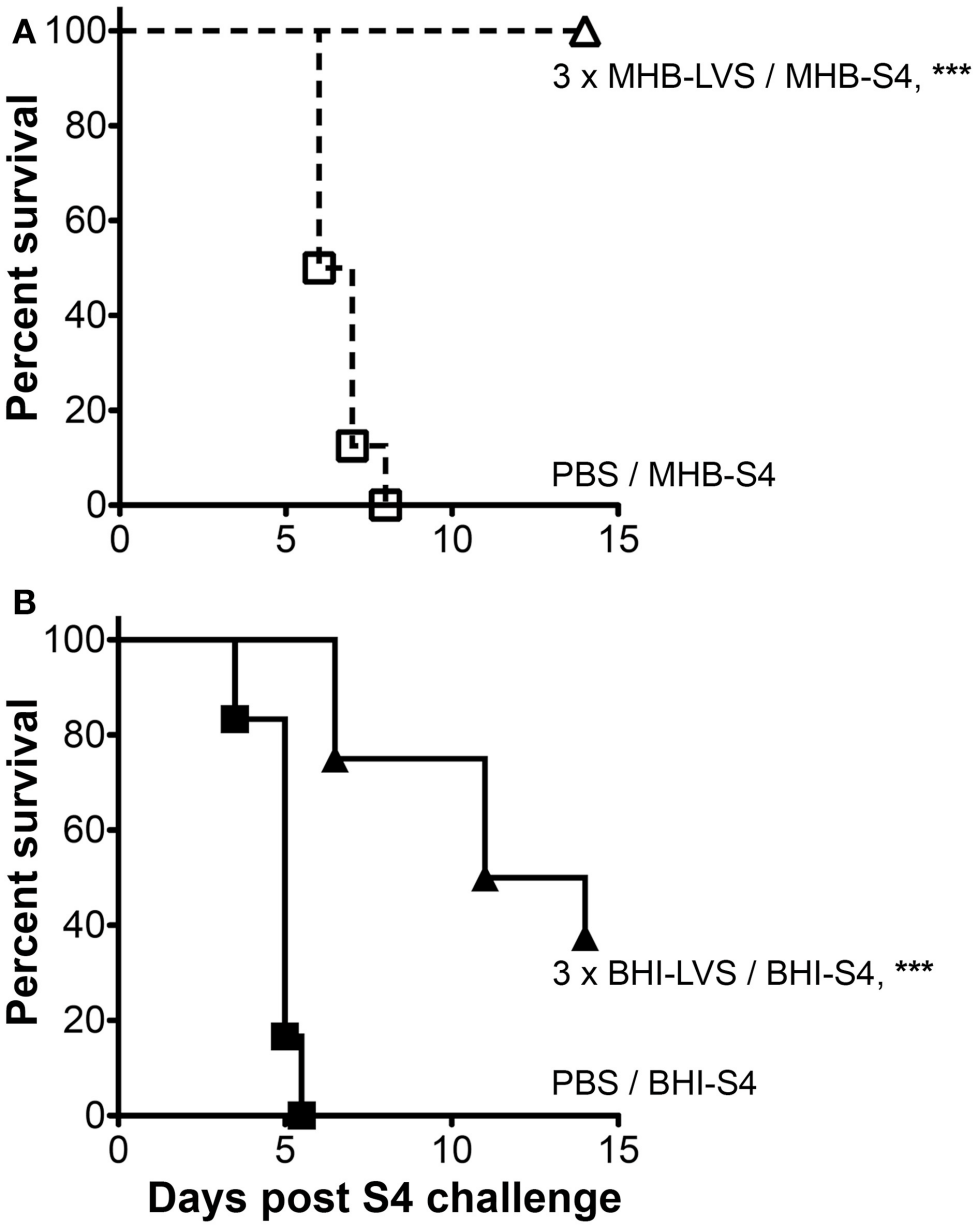
The outcome of tularemia vaccination trials can be influenced by differential growth. Survival of BALB/c mice vaccinated three times with PBS (squares) or live *Ft* LVS (triangles) prior to challenge with *Ft* S4. In **(A)** all *Ft* were grown in MHB (open symbols); in **(B)**, all *Ft* were grown in BHI (closed symbols). Asterisks indicate significant differences between PBS- and LVS-immunized groups that were identically-challenged. Actual challenge doses were 25 CFU of MHB-*Ft* S4 **(A)** and 34 CFU BHI-*Ft* S4 **(B)**. Actual *p* < 0.0001 **(A)** and *p* = 0.0001 **(B)**. Median survival times (MTS) for LVS-immunized mice were ≥ 14 d **(A)** and 12.5 d **(B)**; for PBS-immunized mice the corresponding values were 6.5 d **(A)** and 5 d **(B)**. The survival data from 30 mice used in two independent experiments with 6–8 mice/group are represented as Kaplan–Meier survival curves with statistical analysis by the Log-rank (Mantel–Cox) test.

Our first indication that the latter might be true came inadvertently from independent vaccination trials. For both trials, BALB/c mice were immunized using a prime-boost-boost strategy in which live *Ft* LVS served as the positive control for protection against challenge with *Ft* S4. In one case, the *Ft* strains were grown in MHB; in the second case both *Ft* strains were cultured in BHI. While LVS immunization provided significant protection (*p* ≤ 0.0001) against S4 challenge in both cases, protection for the MHB-based trial was absolute (100%) but only partial (38% survival, 12.5 d MST) for the BHI-based trial (Figure 4). The MST for control mice challenged with MHB-*Ft* S4 was 6.5 vs. 5 d for those challenged with BHI-*Ft* S4. While these results were intriguing, differences between the independent trails necessitated further experimentation.

**Figure 5:**
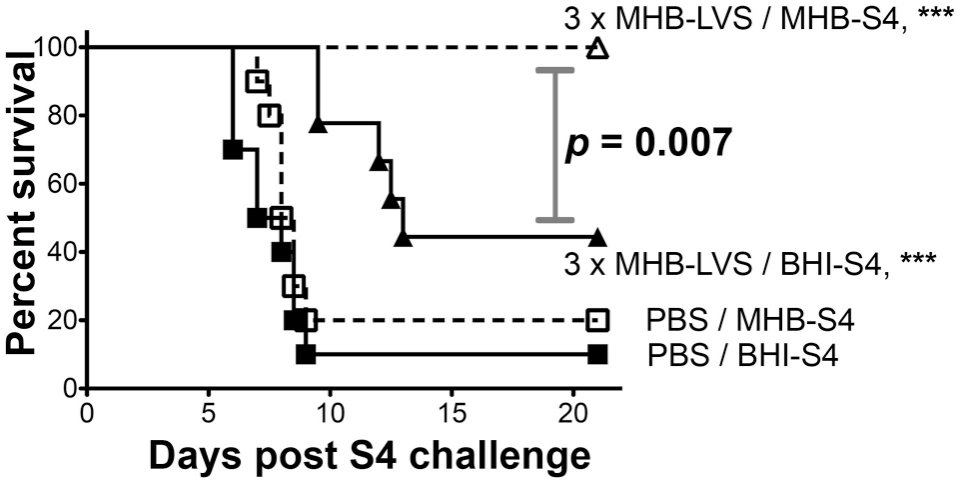
BHI-*Ft* S4 appears more virulent than MHB-*Ft* S4 to mice immunized with MHB-LVS. Survival of C57BL/6 mice vaccinated three times with PBS or live MHB-*Ft* LVS prior to i.n. challenge with 26 ± 2 CFU of *Ft* S4 grown in MHB or BHI. Symbols are as defined in Figure 4; survival differences between immunized mice challenged with MHB-S4 and BHI-S4 are indicated by the stated *p*-values. Asterisks indicate significant differences between groups (PBS or LVS) that were identically-challenged; actual p valves are *p* < 0.0001 (MHB-S4) and *p* < 0.0001 (BHI-S4). All significant differences are indicated. MTS values are ≥ 21 d (LVS/MHB-S4), 13 d (LVS/BHI-S4), 8.3 d (PBS/MHB-S4), and 7.5 d (PBS/BHI-S4). The survival data from 40 mice (10 mice/group) are represented as Kaplan–Meier survival curves with statistical analysis by the Log-rank (Mantel–Cox) test.

**Figure 6:**
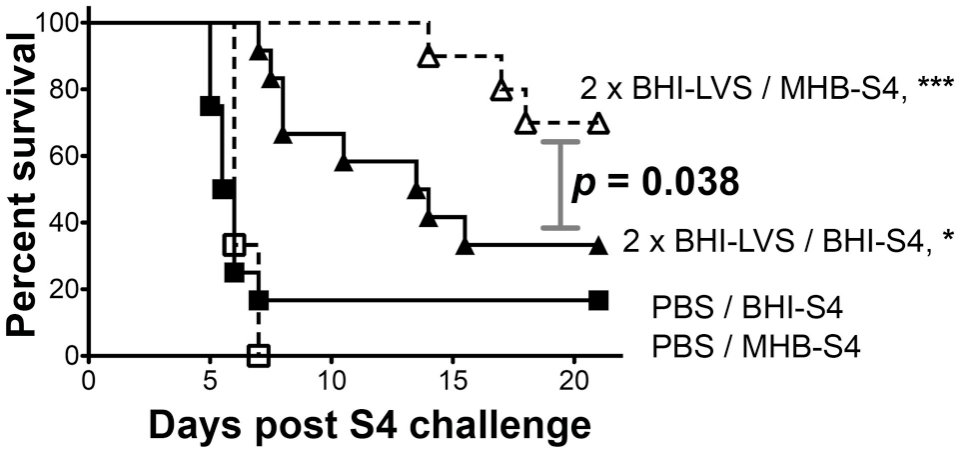
BHI-*Ft* S4 appears more virulent than MHB-*Ft* S4 to mice immunized with BHI-LVS. Survival of C57BL/6 mice vaccinated twice with PBS or live BHI-*Ft* LVS prior to challenge with 29.5 ± 2.5 CFU of *Ft* S4 grown in MHB or BHI. Symbols are as defined in Fig 4; survival differences between immunized mice challenged with MHB-S4 and BHI-S4 are indicated by the stated *p* values. Asterisks indicate significant differences between groups (PBS or LVS) that were identically-challenged; actual *p-*values are *p* < 0.0001 (MHB-S4) and *p* < 0.017 (BHI-S4). All significant differences are indicated. MTS values are >21 d (LVS/MHB-S4), 13.8 d (LVS/BHI-S4), 6 d (PBS/MHB-S4), and 5.8 d (PBS/BHI-S4). The survival data from 46 mice (10–12 mice/group) are represented as Kaplan–Meier survival curves with statistical analysis by the Log-rank (Mantel–Cox) test.

To assess more rigorously the impact of differential cultivation on *Ft* vaccination, we set up a large-scale trial in which mice vaccinated with live MHB-*Ft* LVS in a prime-boost-boost strategy were challenged with either MHB- or BHI-grown *Ft* S4. For these experiments, we used C57BL/6 mice, which are harder to protect against challenge with *Ft* S4 (Twine et al., 2012a), as a highly stringent test of immunization efficacy. For LVS-immunized mice, we again observed complete (100%) protection against challenge with MHB-*Ft* S4 (Figure 5), which was significantly (*p* = 0.007) higher than the survival observed for mice challenged with BHI-*Ft* S4 (44% survival, 13 d MST). Survival of control mice was not significantly different between the MHB-*Ft* S4 group (8.3 d MST) and the BHI-*Ft* S4 challenged group (7.5 d MST). In the former experiment, the *Ft* LVS used for vaccination had been grown in MHB raising the specter that survival among the mice challenged with MHB-*Ft* S4 might have been the result of a more specifically tailored, vaccine-induced immunity. To test this notion, we assessed the ability of BHI-*Ft* LVS to protect against challenge with MHB- and BHI-grown *Ft* S4. C57BL/6 mice were primed and boosted a single time with live BHI-*Ft* LVS. Contrary to the notion of immunogen-tailoring, we again observed that immunized mice survived challenge with MHB-*Ft* S4 (70% survival, undefinable MST) significantly better (*p* = 0.038) than those challenged with BHI-*Ft* S4 (33% survival, 13.75 d MST) (Figure 6). Survival of PBS-immunized control mice was not significantly different (*p* = 0.398) between the groups challenged with MHB-*Ft* S4 (6 d MST) and those challenged with BHI-*Ft* S4 (5.8 d MST).

Collectively, our vaccination studies demonstrate that challenge with BHI-*Ft* S4 provides a more stringent test of vaccine efficacy than challenge with MHB-*Ft* S4.

## Discussion

Several years ago we began characterizing broth- and MΦ-grown *Ft* with the goal of identifying culture conditions that would yield “host-adapted”-like bacteria. Our rationale was that these *Ft* could be used with greater confidence in efforts to (i) identify vaccine Ag candidates expressed during infection and/or (ii) unravel details of the host-pathogen interaction that are operable during infection. In previous studies, using a limited cadre of ~10 metrics that discern MHB-*Ft* from MΦ- or mouse-grown *Ft*, we concluded that *Ft* LVS and S4 grown in BHI roughly mimic the phenotype of the mammalian, infection-derived pathogen (Hazlett et al., 2008; Zarrella et al., 2011). Here we used ~100-fold more data to extend and further characterize the phenotypes of MHB-, BHI-, and BCA- grown *Ft*. By comparing these FCs to those previously reported for infection of cells or mice, we can reasonably conclude that the differences observed between *Ft* grown in MHB and BHI are most similar to that of LVS grown in MHB (or BCA) and host cells for 8 and 48 h (*R*^2^ = 0.3 and 0.37). These correlation coefficients are significantly higher than that of our data fit to LVS grown to stationary phase in CDM (*R*^2^ = 0.17). Similarly, our data best fit that of S4 grown in MΦs for 12 h (*R*^2^ = 0.18), which is higher than the results for stationary phase CDM-grown S4 (*R*^2^ = 0.10). Presumably, growth within host cells elicits changes in *Ft* that are not fully recapitulated by growth in CDM but are more closely (though not perfectly) mimicked by growth in BHI. While comparing FCs across distinct “omics” platforms and between different laboratories presents some limitations (which likely lowered the observed *R*^2^-values) the over-arching notion that BHI-*Ft* are more “host-adapted”-like than MHB-*Ft* appears largely intact following interrogation of ~1,000 data points.

While the primary thrust of our proteomics approach was to test the notion that BHI-grown *Ft* are more similar to infection-derived *Ft*, our data also allows for reasonable interpretation of likely regulatory processes and players. In *Ft*, two highly similar proteins, MglA and SspA, act in concert with FevR, a DNA- and ppGpp-binding protein, to engage the RNA polymerase complex and activate transcription of the MglA/SspA/FevR regulon (which includes *fevR* but not *mglA* or *sspA*) in response to elevated levels of ppGpp (Charity et al., 2007, 2009; Faron et al., 2013; Rohlfing and Dove, 2014; Ramsey et al., 2015). The majority of the FevR regulon have been shown to be up-regulated in one or more infection models, necessary for virulence (Baron and Nano, 1998; Santic et al., 2005, 2007; Brotcke et al., 2006; Tempel et al., 2006; Su et al., 2007; Bonquist et al., 2008; Sammons-Jackson et al., 2008; Barker et al., 2009; Buchan et al., 2009; Dean et al., 2009; Schmerk et al., 2009; Ahlund et al., 2010; McCaffrey et al., 2010; Broms et al., 2011, 2012a; Straskova et al., 2012; Robertson et al., 2013, 2014), and, as shown here, are more abundant in BHI-*Ft* (Table 1 and Table S1). Charity et al. have used genetic approaches to show that activation of the FevR regulon requires ppGpp generated by the activity of RelA and/or SpoT (Charity et al., 2009). Dean and Faron have used serine hydroxamate to induce amino acid starvation (Dean et al., 2009; Faron et al., 2013) and observed that this starvation causes ppGpp levels to rise by 2- to 3-fold with a corresponding 2- to 3-fold increase in *iglA* promoter activity (Faron et al., 2013). Consistent with the impact of serine hydroxamate, we found the absence of casamino acids induced an average FPI protein induction of 2.2-fold and a 2.6 induction of FevR (Table 1). These values are lower than the full FPI induction reported for *Ft* grown in MΦ for 8 h (ave-FPI 4.6, FevR 6.9) and what we observed between BHI- and MHB- grown *Ft* (ave FPI-5.3, FevR 5.0). These data suggest that while amino acid limitation alone can induce the FevR regulon 2- to 3-fold, additional, possibly nutritional, signals contribute to full FPI induction. Severe nutrient starvation and induction of the stringent starvation response (mediated by RelA/SpoT and ppGpp) results in decreased abundance of ribosomes. Of the 47 ribosomal proteins for which we had robust FC data, 24 were statistically less abundant in BHI-*Ft* compared to MHB-*Ft* (Table 1 and Table S2) Among these 47 proteins, 8 were reduced in BHI compared to BCA; 12 and 32 are reportedly reduced after 4 and 8 h respectively of growth in MΦ. Thus in our data, as well as that of Bent for LVS (Bent et al., 2013) and Lenco for LVS and S4 (Lenco et al., 2009), FPI induction appears to correlate with ribosomal protein reduction. Intriguingly, siderophore and pantheonate synthesis proteins are elevated in BHI-*Ft* compared to MHB-*Ft* but not between BHI- and BCA-*Ft* (Table 1) possibly indicating that the iron and pantheonate present in MHB act as additional nutritional cues guiding the stringent starvation response. Up regulation of these genes *in vivo* and their contributions to virulence have been reported previously (Deng et al., 2006; Sullivan et al., 2006; Lenco et al., 2007, 2009; Ramakrishnan et al., 2008; Wehrly et al., 2009; Bent et al., 2013; Miller et al., 2013; Thomas-Charles et al., 2013).

In the course of our analysis we also noted media-dependent changes in protein abundance that mirror changes observed during infection, but for proteins that have not been shown to be dependent on the major regulatory players such as MglA/SspA/FevR, or PmrA. Such proteins include the 12 FPI co-regulated proteins such as Dgt (FTL1503) identified by Lenco et al. (2009) as well as several proteins that are repressed by growth in BHI and mammalian cells such as GuaB (FTL1478) and MetK (FTL1739) (Table 1 and Table S1). The regulatory mechanisms governing expression of these MglA/SspA/FevR/PmrA-independant proteins, many of which appear to be growth phase insensitive (Lenco et al., 2009), remain to be elucidated. Another group of proteins worth noting are those that have been shown to be up-regulated during infection but were not up-regulated in BHI-*Ft*. The primary examples of this are several ATP synthases (FTL1794-1798) and TCA enzymes (FTL1783-1786) which we detected as media-non-responsive but have been shown in LVS and S4 to be down-regulated during infection of cells and mice respectively (Twine et al., 2006a; Bent et al., 2013). Thus, BHI-*Ft* are good, but not perfect, mimics of host-adapted *Ft*.

Given the differences in protein abundance, it is not surprising that host-adapted *Ft* have an initial growth advantage *in vivo* that manifests as a slightly shorter MST of naïve animals infected with BHI-*Ft* (Hazlett et al., 2008; Zarrella et al., 2011). Our finding that these differences in apparent virulence are magnified in the context of pre-existing specific immunity further supports the notion that the growth status of *Ft* is an important factor to be considered in tularemia research. For example, in three separate experiments, challenge of LVS-vaccinated mice with MHB-*Ft* S4 revealed survival rates of 100, 100, and 70%; the corresponding survival rates for mice challenged with BHI-*Ft* S4 in the same experiments were 38, 44, and 33%. Clearly, the status of a *Ft* challenge inoculum at the time of administration can have a profound impact on experimental outcomes in tularemia vaccine research.

At this time, we cannot state with singular certainty why MHB-*Ft* S4 appears less virulent to immunized hosts than does BHI-*Ft* S4. However, we suspect that this outcome stems from the transient, phenotypic differences between MHB- and BHI-*Ft* that exist immediately upon host-challenge and prior to complete host adaptation. Broadly speaking we can envision three inter-related properties (reduced capsule, reduced structural integrity, and reduced VF expression) that likely weaken a MHB-grown, S4 challenge inoculum sufficiently to allow pre-existing specific immunity to gain the upper hand. First, MHB-*Ft* LVS and S4 have less capsular material relative to their BHI-grown counter-parts (Zarrella et al., 2011). This capsule reduces complement deposition, which would limit losses of challenge bacteria to alternatively-activated complement upon entry into the mammalian milieu (Zarrella et al., 2011). *Ft* capsule also limits access of specific Ab to multiple *Ft* Ags (Zarrella et al., 2011). Reduced Ab binding would have two salient outcomes: (i) bacterial losses due to classical complement activation would be minimized and (ii) Ab-mediated phagocytosis through activating Fc-receptors would be reduced. This capsule also limits the access of *Ft* ligands to TLR2—an activating receptor responsible for MHB-*Ft*-mediated production of T_H_1-proinflammatory cytokines (Zarrella et al., 2011; Singh et al., 2013). Also, we envision that the capsule may directly or indirectly engage a discrete “anti-inflammatory” receptor that also could diminish elicitation of protective cytokine responses. Along these lines, the Bosio group recently reported that exogenous, purified *Ft* capsule was able to dampen MΦ cytokine responses to P3C (a synthetic TLR2 agonist) and to an inflammatory, capsule mutant of *Ft* S4 (Wyatt et al., 2016). Cumulatively, given the well-established role of capsules in promoting bacterial survival, it seems highly likely that differences in survival of immunized hosts would be observed between challenges with (i) bacteria encased in abundant, pre-formed capsule (such as BHI-*Ft*) vs. (ii) those entering the hostile mammalian environment without a full protective coating of pre-made capsule (such as MHB-*Ft*).

The second property of MHB-*Ft* that may contribute to reduced fitness in the face of pre-existing immunity is their compromised structural integrity, which promotes rapid T_*H*_1 pro-inflammatory responses. This pro-inflammatory nature which makes i*Ft* LVS a better immunogen (Kumar et al., 2016) could also make MHB-*Ft* S4 a worse pathogen. Previously, we stained early-log phase MHB- and BHI-grown *Ft* with a fluorescent vital dye mix and analyzed the stained bacteria by flow cytometry. MHB-grown cultures contained five-to-seven times more structurally-compromised (SYTOX green positive) *Ft* than did BHI-grown cultures (Singh et al., 2013). We also found that compromised *Ft* induce potent cellular responses (TNF, IL-1β, and IL-6) as first reported by Peng et al. (2011). The SYTOX-positive MHB-*Ft* (~20% of the total) were comprised of two populations, one that was moderately stained (fluorescence intensities of 10^1^–10^2^) and one that stained intensely (>10^3^). The fluorescence intensity of the SYTOX-positive BHI-*Ft* (3.5% of the total) barely exceeded 10 with no signal greater than ~30. Although not directly tested, we suspect that the severely compromised *Ft* (present exclusively in MHB-grown cultures) are replication-incompetent and act simply as an inflammatory bolus within an MHB-inoculum. The moderately-compromised MHB-*Ft* might be able to replicate in a sufficiently permissive environment (such as a chocolate agar plate or a naïve host) but are hypothesized, in the presence of pre-existing specific immunity, to be rapidly recognized and thus serve as additional inflammatory agonists to engage protective recall responses. As cultures of BHI-*Ft* contain fewer, and less intensely stained, SYTOX-positive bacteria, we envision that a BHI-*Ft* challenge inoculum remains stealthy enough to avoid a rapid recall response by immunized mice thereby allowing for more productive infections.

The third property of MHB-*Ft* that likely promotes susceptibility to vaccine-induced-immunity is under-expression of virulence factors. We found that 25% of the ~190 *Ft* VFs are less abundant in MHB-*Ft*. When a challenge inoculum of MHB-*Ft* enters the relatively permissive environment of a naïve host, a sufficient number of viable bacteria in the inoculum are able to replicate and host-adapt, turning on the VFs in the process. As such, the differences in MST between naïve (or PBS-immunized) mice challenged with MHB- and BHI-grown *Ft* are small (Figures 4–6) (Hazlett et al., 2008; Zarrella et al., 2011). In contrast, upon entry into the less-permissive environment of a vaccinated host, the MHB-*Ft* inoculum still requires time to adapt but now must do so while being targeted by *Ft*-specific immune effectors. Within mammalian hosts, one of the main functions of the bacterium's well-studied VFs—the FPI proteins—is to promote rapid entry into the host cytoplasm where intact *Ft* replicates exponentially (Meibom and Charbit, 2010; Ozanic et al., 2015). We anticipate that an inoculum of MHB-*Ft* likely spends more time being assaulted by extra-cellular immune effectors prior to cytoplasmic replication than a BHI-*Ft* inoculum. Given a strong enough armamentarium of immune effectors, a MHB-*Ft* inoculum might be unable to mount a productive infection whereas a BHI-*Ft* inoculum is less susceptible to, and spends less time dealing with, these effectors resulting in more productive infections.

## Ethics statement

Use of these human sera was reviewed by the Albany Medical Center Committee on Research Involving Human Subjects Institutional Review Board and deemed exempt on 4-28-2014. Exemption was granted on the basis that the samples were de-identified and came from previously banked collections (i.e., no fresh sera draws were performed for work in this manuscript).

## Author contributions

Conceived and designed the experiments: KK, EB, TS, SJK, EG, DR, KH. Performed the experiments: KH, SR, KK, DW, BF, TZ, SK, RS, AS, PN, CB, KH. Analyzed the data: KK, CB, EB, TS, SJK, EG, DR, KROH. Contributed reagents/materials/analysis tools: EB, TS, SJK, EG, DR, KH. Wrote the paper: KROH.

### Conflict of interest statement

The authors declare that the research was conducted in the absence of any commercial or financial relationships that could be construed as a potential conflict of interest. The reviewer VP and handling Editor declared their shared affiliation, and the handling Editor states that the process nevertheless met the standards of a fair and objective review.
