# Supplementary material for: Differential Growth of Francisella tularensis, Which Alters Expression of Virulence Factors, Dominant Antigens, and Surface-Carbohydrate Synthases, Governs the Apparent Virulence of Ft SchuS4 to Immunized Animals
Source: Front Microbiol. 2017 Jun 22;8:1158. doi: 10.3389/fmicb.2017.01158 (PMC5479911; doi:10.3389/fmicb.2017.01158)
Supplement: Supplementary file 3 [file Image1.PDF]

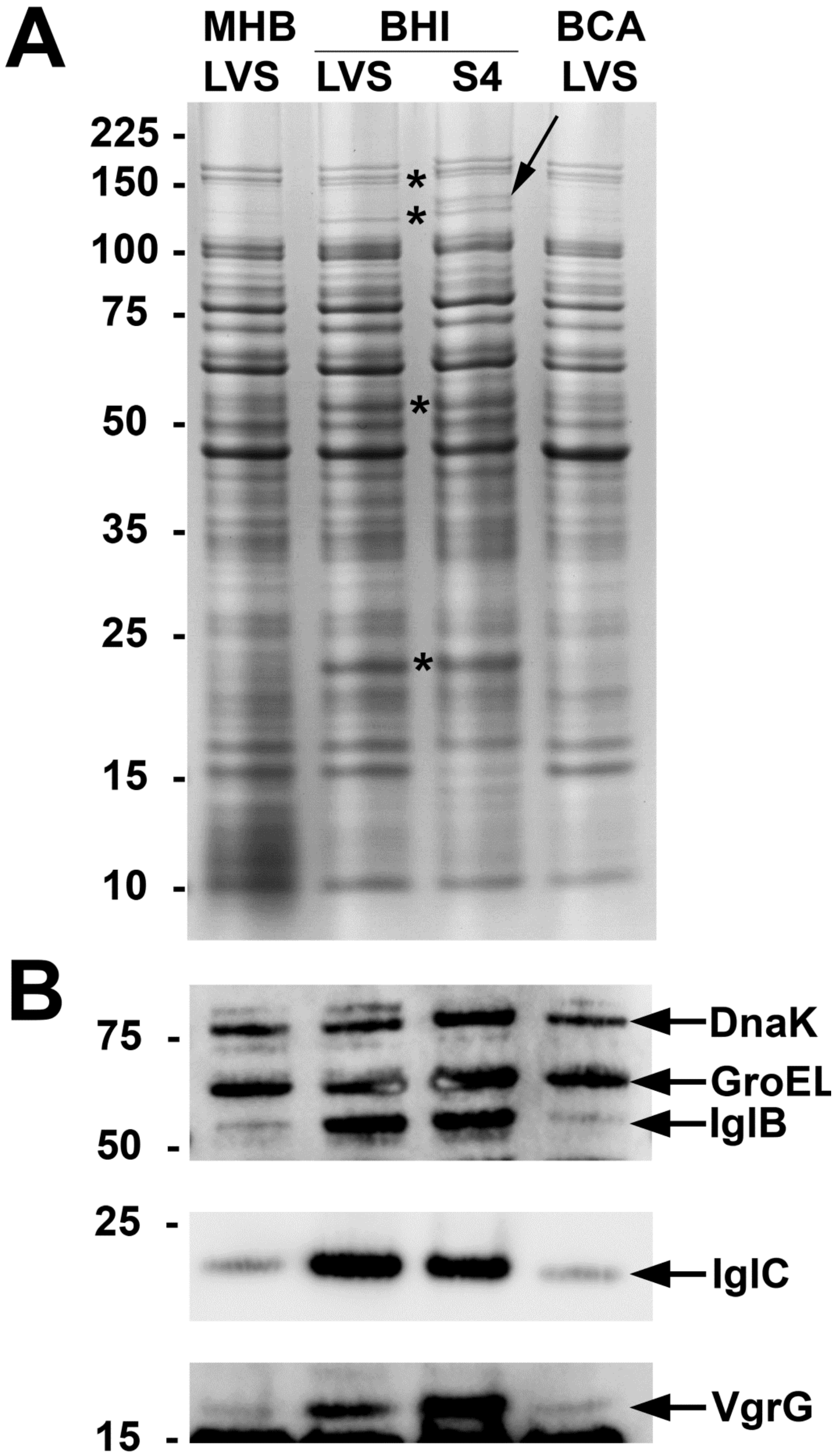

Fig S1. Confirmation of select "omics" data. Ten ug of *Ft* LVS and S4 grown as indicated were resolved by SDS-PAGE and stained with Coomassie blue (**A**) or transferred for western blot analysis (**B**). Asterisks in (A) mark BHI-specific bands common to *Ft* LVS and *Ft* S4; the arrow in (A) points to a ~135 kDa, *Ft* S4-specific protein. Membranes in (B) were probed with a cocktail of murine monoclonal antibodies (mAb) specific for DnaK, GroEL, and IglB, a mAb specific for IglC, or polyclonal rabbit anti-VgrG serum.
