# Supplementary material for: Differential Growth of Francisella tularensis, Which Alters Expression of Virulence Factors, Dominant Antigens, and Surface-Carbohydrate Synthases, Governs the Apparent Virulence of Ft SchuS4 to Immunized Animals
Source: Front Microbiol. 2017 Jun 22;8:1158. doi: 10.3389/fmicb.2017.01158 (PMC5479911; doi:10.3389/fmicb.2017.01158)
Supplement: Supplementary file 4 [file Image2.PDF]

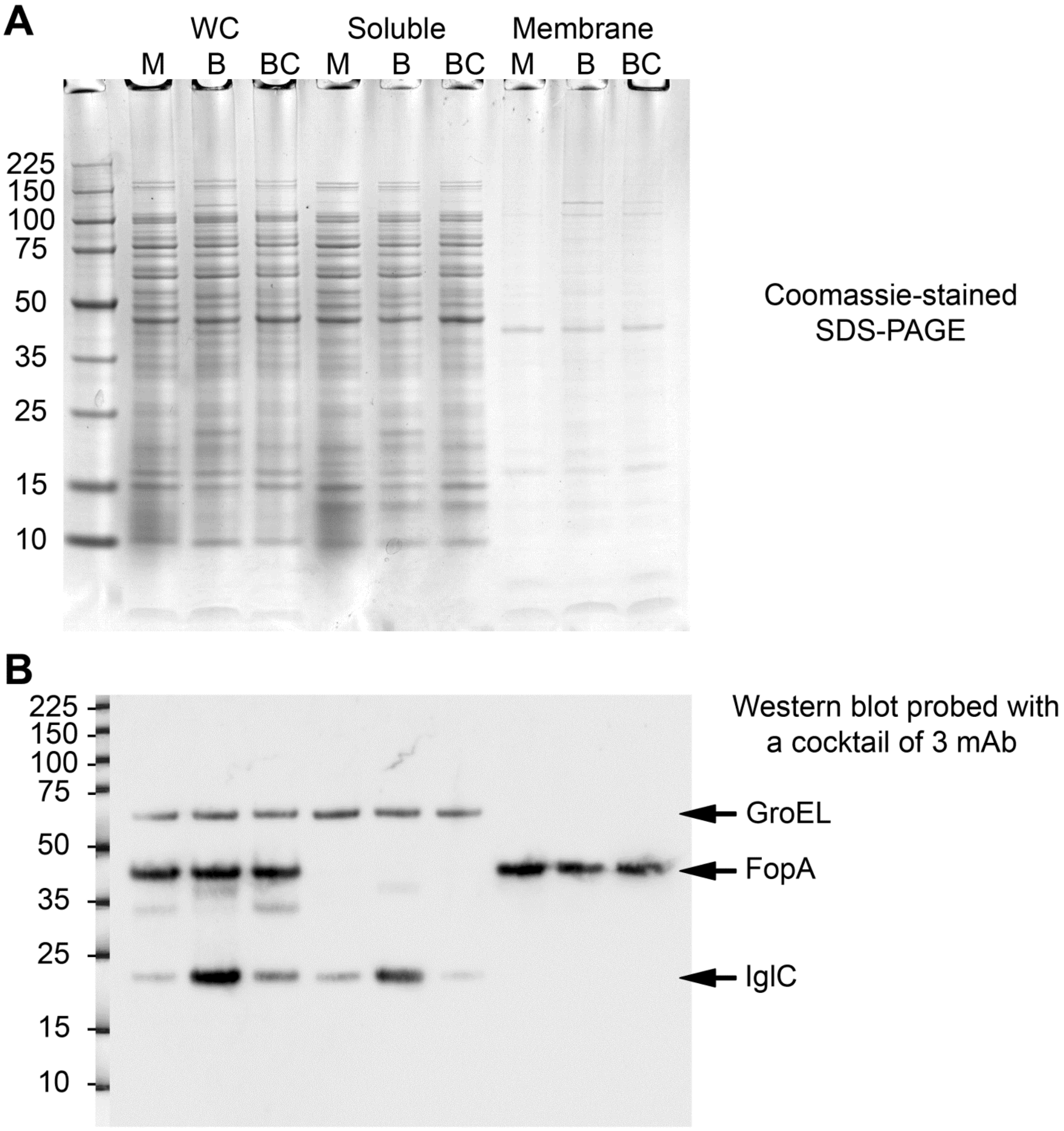

Fig S2. Confirmation of loading and fractionation efficiency. Whole cells (WC) lysates of *Ft* LVS grown in MHB (M), BHI (B), or BCA (BC) were partitioned into soluble and membrane fractions prior to SDS-PAGE (panel **A**) and western blot analysis (panel **B**). Each lane was loaded with material derived from 1*10e8 *Ft*. The membrane in B was probed with a cocktail of mAbs specific for GroEL, FopA, and IglC.
